# Digital technologies as solutions to China’s aging population: a systematic review of their opportunities and challenges in rural development

**DOI:** 10.3389/fpubh.2024.1416968

**Published:** 2025-01-15

**Authors:** Yue Ming, Yimin Li, Yun Liu

**Affiliations:** ^1^Digital Art Industry Institute, Hubei University of Technology, Wuhan, China; ^2^School of Art & Design, Hubei University of Technology, Wuhan, China; ^3^Department of Product Design, Faculty of Fine Arts and Design, College of Chinese & ASEAN Arts, Chengdu University, Chengdu, China

**Keywords:** smart aging, older people, digital technologies, aged people, rural development

## Abstract

**Introduction:**

Differences exist in the rate of aging between individuals residing in urban and rural areas in China, with rural areas experiencing a more pronounced impact. Smart aging represents a prevalent future trend in this regard, though its development will inevitably face challenges. However, studies focusing on rural areas are scarce. Future models of aging in less developed regions, including rural areas and townships, are expected to integrate and draw inspiration from the smart aging paradigm.

**Methods:**

We present a systematic review of the current application of digital technologies in caring for older people in rural areas. We conducted extracted and screened 26 articles out of 823, sourced from five databases: Web of Science, IEEE Xplore, Engineering Village, PubMed/MEDLINE and CNKI.

**Results:**

The articles focus on digital technologies that cater to the daily life, medical care, spiritual comfort, and cultural entertainment needs of rural older people. In our review, we focused on four aspects of digital technology: mobile applications (apps), websites and platforms, mobile devices and terminals, and VR technologies or other unspecified technologies.

**Discussion:**

We found that current digital technologies for smart aging have room for improvement in meeting the recreational and mental comfort needs of older adults. Digital technologies are predominantly applied to the smart community/rural and smart healthcare sectors, with limited applications in the smart home sector. Future studies should explore smart-home older adults care services to address digital cognitive barriers faced by older adults.

## 1 Introduction

In recent decades, the world’s population has experienced changes in age composition undergone changes in the form of declining growth rates and aging (1). According to a report from the United Nations World Population Prospects, by the mid-century, there will be 1.5 billion people aged 65 years old in the world, constituting approximately one-sixth of the total population (2). In China, which is a typical developing country, the proportion is even higher: by 2050, there will be approximately 400 million people aged 65 years or over, representing approximately one-third of the country’s total population (3). The World Health Organization (WHO) defines people aged 60 and above as older adults (4). The United Nations considers individuals 65 and older to be older adults (3), while Mainland China uses the age of 60 and above to plan medical and other services for older adults (5).

Globalization has wrought profound changes in rural areas, influencing social, economic, and environmental factors (6). An escalating flow of production elements from rural to urban locales has exacerbated the pressing issues of population decline and economic regression in rural regions. Notably, in China, disparities in the aging demographic are more pronounced in rural areas than in urban areas. Common challenges in rural settings include inadequate infrastructure and a dearth of medical resources, compounding the hurdles posed by an aging population. Seeking economic and personal advancement opportunities, numerous rural residents opt to migrate to large urban centers, leaving their older relatives behind, thereby giving rise to phenomena, such as “hollow villages” and “empty-nest older households.” Consequently, the patterns of population aging in China have manifested differently in urban and rural landscapes. Addressing the health and safety challenges of older adults in rural China requires a scientific and sustainable approach. Such endeavors not only aim to rectify the challenges faced by rural communities but also enhance the overall welfare of the entire populace (7).

Research in fields such as smart older adults care and digital technologies has steadily expanded, showing significant advancements (8, 9). Increasing evidence indicates that the use of sensors, wearable devices, artificial intelligence-powered telemedicine, and mobile and cognitively supported interactive robotics has led to a notable enhancement in the quality of life of older individuals. These technologies offer medical assistance and to promote independence, thereby contributing to an improved quality of life for older adults. Furthermore, digital technologies, including telemedicine and virtual reality, are being employed to address the healthcare needs and social isolation of aging populations, particularly in rural areas. Collectively, these studies underscore the potential for integrating digital solutions into the care of older adults, with the aim of enhancing overall well-being and supporting rural development (10). Despite the overall progress, studies focusing specifically on rural areas are relatively scarce. However, some existing research has begun to explore this domain. For example, studies have explored the implementation of telemedicine services in rural regions, demonstrating improvements in healthcare access and patient outcomes (11). Additionally, a previous study evaluated whether walking programs using wearable devices can impact the physical activity and health outcomes of rural older adults (12). The studies primarily focus on health-related applications, with less attention given to addressing the comprehensive needs of rural older adult individuals, such as social engagement, leisure activities, and mental well-being.

Smart older adults care has become a natural and inevitable choice for the future older adults care. In the forthcoming years, older adults care in underdeveloped regions, including rural and township areas, is expected to integrate and derive insights from the models of smart older adults care. Chanjun Liu and her colleagues have advocated for a shift in focus toward potential users of smart older adults care in rural and township settings. They have emphasized the need for forward-looking research on user needs, aiming to uncover the authentic value demands of the older population that may be overshadowed by technological considerations (13).

Through a systematic literature review, we identified the advantages of the smart older adults care industry and formulated the following research questions:

1. What is the current status of digital technology applications for aging in rural China?
2. How do existing smart aging technologies address the needs of rural older residents, and which specific needs are overlooked?
3. What is the future of these technologies?

Therefore, in this review, we aim to explore the current application status of digital technologies to smart older adults care in rural China. We focus on older people in rural areas and explore their neglected needs for smart aging solutions. Ultimately, we investigated the directions of future development of these technologies, aiming to provide sustainable and innovative solutions for the challenges posed by rural aging.

## 2 Materials and methods

### 2.1 PRISMA statement

**Figure 1:**
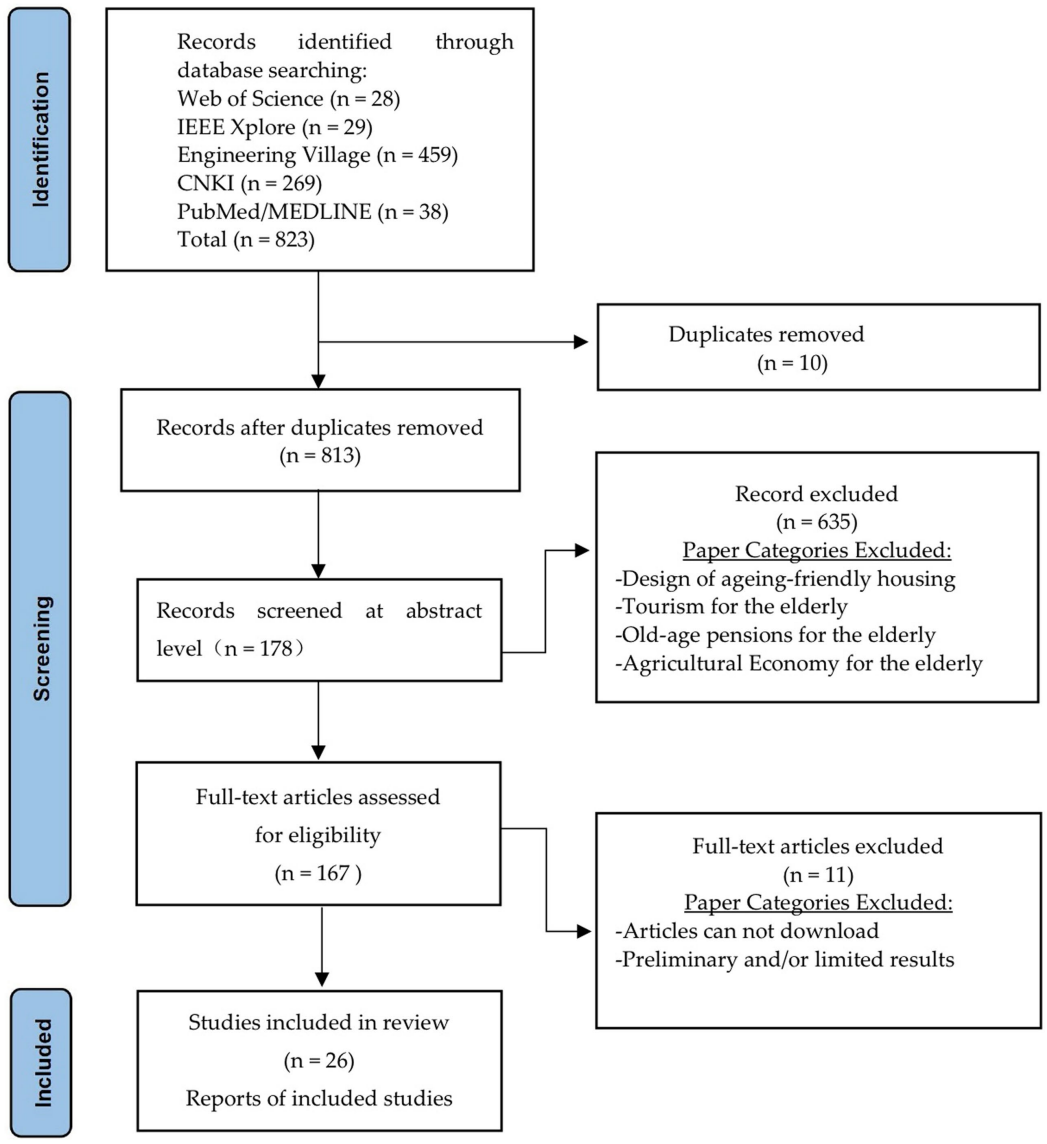
Schematic illustration of the literature search.

The primary aim of this systematic review was to identify relevant literature on the application of digital technologies in smart older adults care within the context of rural development in China. Our review followed the Preferred Reporting Items for Systematic Reviews and Meta-Analyses (PRISMA) guidelines (14). Given the focus of our research on technology-related social issues in China, we selected the following databases: IEEE Xplore and Engineering Village (EI), which are literature databases for engineering; Web of Science, a comprehensive database of high-quality journals from around the globe; PubMed/MEDLINE is a database of biomedical and health science literature; and China National Knowledge Infrastructure (CNKI). We conducted a comprehensive search in these databases, which was divided into four steps: (1) identification, (2) screening, (3) eligibility assessment, and (4) data extraction. Figure 1 shows the screening strategy using the PRISMA flowchart.

### 2.2 Search strategy and information sources

We focused on the application of digital technologies in response to the challenges posed by the aging population in rural China, with a specific emphasis on three critical domains: (1) smart aging, (2) rural areas, and (3) digital technologies.

The search strings employed for various databases were as follows: (“smart aging” OR “smart ageing” OR “intelligent ageing” OR “intelligent aging”) AND (“village” OR “townships” OR “rural area” OR “rural” OR “countryside”) AND (“China”). The time period of our searches spanned from the inception of the databases to April 10, 2024, with language limitations set to Chinese and English. The search results were meticulously sorted based on relevance. Additionally, we delved into gray literature sources, encompassing conference papers, government reports, and working papers, to comprehensively gather pertinent articles. This exhaustive search process culminated in a total of 823 identified studies.

### 2.3 Screening and eligibility

Utilizing advanced retrieval techniques, all fields (including the subject, title, abstract, and keywords) in the database were searched for using the combinations of the mentioned terms. Articles meeting the criteria outlined in Table 1 were selected based on various standards. The inclusion criteria were as follows: (1) discussing the current application status of smart older adults care to rural China; (2) articles available in both Chinese and English; (3) addressing the needs of the older adult regarding smart older adults care.

**Table 1:** Inclusion and exclusion criteria.

Criterion	Inclusion	Exclusion
1	Digital technology for aging in China in rural areas	Duplicates from different databases
2	English and Chinese-written articles	Published in other languages
3	Article on the analysis of the needs of the older adults for smart aging	Research that is not related to digital technologies for smart aging, is not in a rural area, or focuses on something else

After retrieval, all the search results were downloaded into reference management software, and duplicate content was removed. Subsequently, a comparative study was conducted on the application of smart older adults care to rural China. Initially, we conducted a relevance assessment on the abstracts of all articles, excluding those related to aging studies in agricultural economics, housing design, tourism, and finance. Thereafter, the remaining abstracts were assessed based on the inclusion and exclusion criteria presented in Table 1.

### 2.4 Data extraction

**Figure 2:**
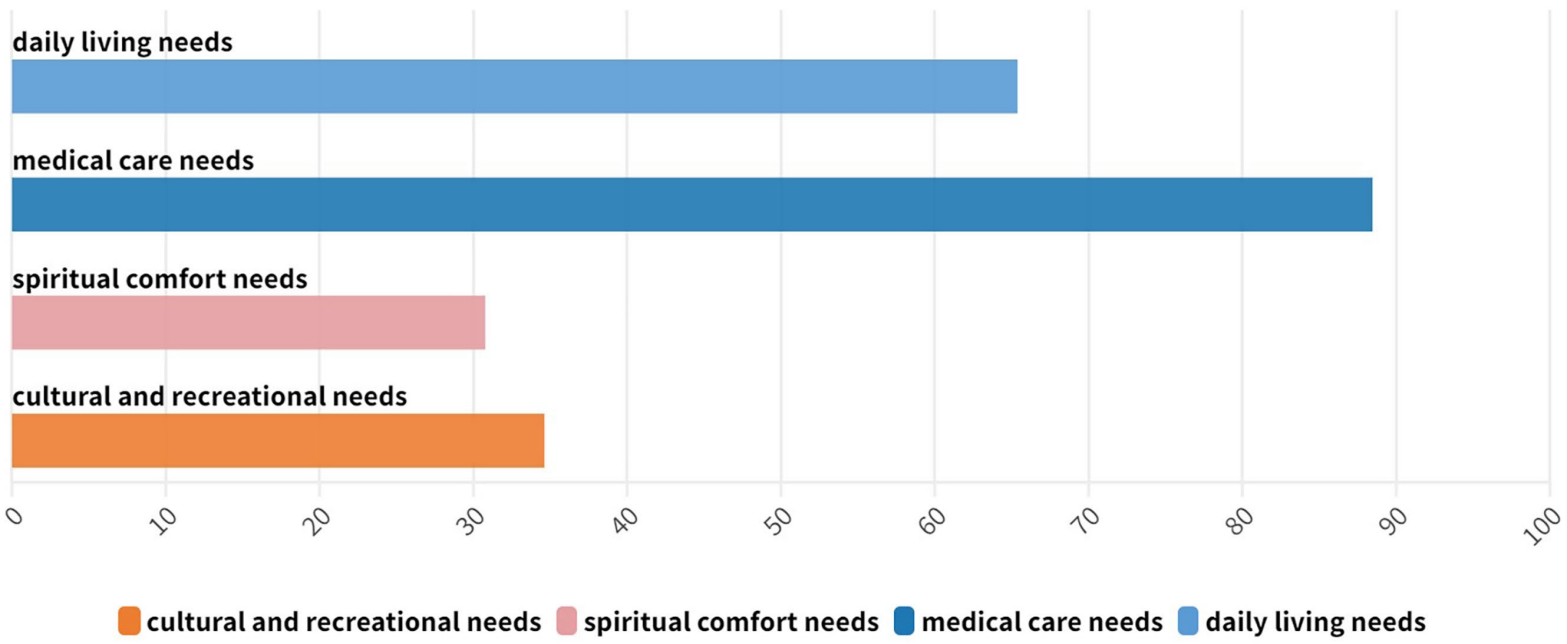
Proportion of literature on digital technologies addressing older adults’ needs.

We reviewed the final list of selected articles against the inclusion and exclusion criteria to confirm their appropriateness for screening. Supplementary Table 1 lists the current status of smart aging applications in rural China, including smart technology categories and strategies. Twenty-six documents were screened and classified into four categories, as shown in Figure 2.

## 3 Results

### 3.1 Paper selection

The article was divided into four sections, providing a review of digital technologies that address the daily living, healthcare, psychological support, and cultural entertainment needs of older people. Approximately 70% (65.38%) of the requests were related to daily living needs, whereas almost 90% (88.46%) of them were related to medical care requirements. Over 30% (30.77%) of the requests were related to spiritual comfort, and approximately one third (34.62%) off them were related to cultural and recreational needs. Table 2 extracts the key technologies for the four themes.

**Table 2:** Key technologies to meet the four categories of needs.

Type of demand	Key technologies and solutions	Reference
Daily living needs	1. Intelligent platforms and devices (real-time service requests, data cloud platforms and smart terminal integration) 2. One-touch connectivity system (integration of landline and mobile devices) 3. Artificial Intelligence (AI) facial recognition, smart displays, infrared temperature sensors, smart robots 4. Integrated service platform combining medical care resources and wearable devices, intelligent information management platforms 5. Virtual Reality (VR) and 3D technologies 6. Smart environmental monitoring technologies	(15, 16, 18–30, 42)
Medical care needs	1. Automatic sign detection, telemedicine, health testing, health counseling, emergency rescue system (including wireless emergency alarm, GPS positioning, intelligent health monitoring) 2. Integrated health management platform (combining wearable devices, remote monitoring, smart devices, smart medical applications, supporting real-time monitoring and health data sharing) 3. Intelligent medication management system (web-based management, age-friendly design) 4. Data sharing and remote monitoring platform (combining health data monitoring, emergency warning function) 5. Internet hospital and remote expert support network	(15–19, 21–27, 30–37, 40, 42)
Spiritual comfort needs	1. Intelligent older adults information platform (push daily care information, weather reminder, companion dialog, psychological counseling) 2. AI interactive smart audio (e.g., Tmall Genie, Xiaodu Audio, etc.) 3. Emotion-regulating robots, companion robots (can recognize and respond to the emotions of the older adults) 4. Landscape node design (combining AI technology and natural landscape to meet the psychological needs of rural residents)	(16, 21, 22, 24, 29, 32, 33, 37)
Cultural and recreational needs	1. Intelligent audio devices and short video system to support square dance activities (recording and sharing interactive videos) 2. Intelligent older adults information system(IoT sensing and data sharing platform to support social needs) 3. Entertainment robots (providing activities such as singing and dancing) 4. Elderly portrait system (data-driven personalized activities such as emotional communication and learning)	(15, 17, 19, 21, 22, 24, 27, 28, 33)

### 3.2 Addressing the daily living needs of older people

With population aging and an increase in the demand for diverse older adult services, the need for high-quality older adults care services at multiple levels is urgent (15). Daily living services are one of the most fundamental older adult services (16) and can be divided into three categories: daily safety needs and day care needs and those that address both of these categories (15).

There are 17 pieces of literature that have addressed the use of digital technology to meet the daily living needs of older people.

For daily safety needs, Shaoxing City has installed a one-button connection between one-key connection landlines and mobile devices for those aged 70 or above in the rural community. This system, featuring easy-to-use buttons, is designed to facilitate prompt assistance (17). Furthermore, Xiangxin Li’s study on remote “hollow villages” suggests that implementing smart service platforms, combined with wearable devices and free “one-touch” senior phone services, can significantly improve the quality of life and safety of the older adults in these areas (18). This is further supported by Zhao Jing’s study (15), which argues that integrating data cloud platforms and smart terminals with building intercom systems and urban–rural security networks can enhance safety and enable real-time monitoring (15). However, the adoption of these technologies faces challenges due to inadequate infrastructure in rural areas and the need for technological adaptations.

For day care needs, several studies have proposed innovative solutions involving artificial intelligence (AI) and robotics. Yujing Gu’s (19) design proposal includes the implementation of an artificial intelligence face recognition system at community entrances and exits, along with smart displays equipped with infrared temperature sensors and weather information for pedestrian and vehicular traffic. Furthermore, Lichun Yang and Yujing Gu (19, 20) identified six distinct functions of intelligent robots that integrate various technologies, such as big data and sensor technologies. These include mobility-assistance robots, daily care robots, and intelligent delivery vehicles, which support daily tasks like helping mobility-impaired older adult individuals with activities such as standing, walking, and sitting (19, 20). However, it should be noted that these products have minor shortcomings, particularly in light control or voice command functionalities.

Additionally, there are instances wherein both of these requirements are fulfilled concurrently, thereby enhancing the overall efficacy of older adult services through the utilization of a technologically facilitated integrated platform (21, 22). The Jiangsu Province has implemented an integrated online and offline senior care service system (23), which employs virtual reality (VR), three-dimensional technology, and integrated display technology to present data on senior care services and provide online and door-to-door services. Similarly, the Wuzhen Home-based Intelligent Nursing Service Center employs an intelligent information management platform to deliver remote services via mobile applications and the Ali Cloud (24–26). This concept was also explored by Ruifang Zhang and Weiwei Wei in their study (16, 27), in which the platform was integrated with a mobile application, enabling family members to arrange services for their older adult relatives, thereby enhancing accessibility and convenience.

Furthermore, the integration of technology has the potential to enhance the surrounding environment. It has been demonstrated that the implementation of intelligent environmental monitoring technologies can markedly enhance the quality of life for older individuals by guaranteeing the maintenance of hygienic, secure and comfortable living environments (28, 29). Technologies such as “Internet+” assist in maintaining the ecological health of older adults care facilities, which is beneficial to physical and mental health (30).

### 3.3 Addressing the medical care needs of older people

Rural smart care for older adults provides a range of older adults care services, with medical care being the most important. This service includes automatic detection of the physical indicators of older people, online remote medical care, emergency assistance, health testing, health counseling, and other medical care services (16).

In the realm of digital technology applications for older adult medical care, 22 studies were considered.

The importance of telehealth monitoring systems in ensuring the health and safety of older people, particularly those residing in rural areas and living alone, has been widely acknowledged in academic research (15, 31, 32). In this context, the study by Rui Zhu and Zijie Zeng (18, 33) highlights that these platforms address this issue by collecting health data through wearable devices and utilizing cloud computing for remote monitoring, thereby facilitating timely medical interventions.

Emergency response systems represent a further critical application area. In Wuzhen, the deployment of infrared sensors and door alarms enables the detection of falls and the transmission of alerts, while the use of GPS locators enhances the efficiency of rescue operations (24–26). Juxian Sun proposed the concept of non-intrusive systems capable of real-time location tracking and alarm-based emergency responses, thereby illustrating the potential of intelligent devices in remote regions (23).

Nevertheless, concerns persist regarding the reliability, safety, and quality of care provided by telemedicine systems in rural areas. Consequently, implementing robust quality assurance mechanisms has become imperative (18, 34). Simultaneously, the exchange of health data across platforms is viewed as a pivotal step in establishing a unified and comprehensive care network. In response, Yuechi Lu and Wei Weiwei proposed an age-friendly retrofit program that incorporates devices capable of integrating health monitoring with emergency alert systems, thereby enhancing communication between older adults and caregivers (27, 35). Similarly, Yan Duan and his team developed a mobile application that facilitates real-time sharing of heart rate, blood pressure, and other physiological data, addressing this issue by enabling instantaneous data exchange between healthcare providers and family members (36).

Furthermore, numerous studies have highlighted the potential of integrating diagnostic tools, health assessments, and healthcare management within smart healthcare platforms to improve communication between older adults and healthcare providers and to optimize service efficiency. The Elderly Portrait System, developed by Yangming Hu et al., evaluates a broad range of health parameters, including mental status and nutrition (17). Similarly, platforms such as Lingli Chu’s grassroots support system, Zhoushan City’s “Smart Aging” service, and Lehoo’s Smart Ward illustrate how health data integration can enhance service precision and reduce response times (17, 18, 22). However, these advancements also highlight challenges related to data standardization and platform usability. To address this, Jiankang He et al. (37) proposed a user-friendly system that combines a web-based management tool and a client application to enhance the efficiency and accessibility of medication management. The system is tailored to accommodate the diverse needs and skill levels of older adults.

### 3.4 Addressing the spiritual comfort needs of older people

Rural smart home care provides intimate senior care services, including daily care, accompanied conversation, psychological counseling, and internet conversation. These services aim to address the spiritual comfort needs of older adults through digital technology applications (16). Eight studies have been considered within this scope.

The Smart Elderly Information Platform has been demonstrated to provide emotional support and care for the older adults through the provision of features such as daily care information, weather reminders, and clothing advice. Furthermore, the real-time communication with cloud platform staff achieved through voice interactions has been shown to be particularly beneficial for lonely and empty-nested older adult individuals residing in rural areas (16, 27). Furthermore, Rui Zhu incorporated a music therapy component into the platform, with the objective of regulating mood and enhancing sleep quality for older users. This was achieved by providing a selection of popular music with proven benefits for sleep (33).

The incorporation of intelligent devices offers a multitude of pragmatic solutions for the management of emotional wellbeing in the older adult population. Such devices as AI smart speakers, emotion-regulating robots, and companion robots are capable of recognizing and responding to the emotional needs of older adults through simple voice commands, thereby providing tailored psychological support and companionship (20, 35). Furthermore, Guangtao Zhou put forth an intelligent health detection system with the objective of discerning emotional fluctuations in older adults (30).

Furthermore, Yujing Gu put forth the concept of the “Rice Dream Space,” which fuses artificial intelligence and landscape esthetics. Through the utilization of Quick Response (QR) codes within the paddy fields, local villagers are able to access information pertaining to the growth cycle, current status, and projected harvest of the crops. This initiative aligns with the esthetic preferences and psychological attachment of rural populations to natural idyllic environments (19). Despite the numerous possibilities afforded by existing technologies, the field of emotional interaction design remains in its nascent stages. In the future, greater attention should be directed toward the design of products tailored to the emotional needs of empty-nesters, with a particular emphasis on the potential for emotional interaction (38).

### 3.5 Addressing the cultural and recreational needs of older people

Older adults typically have more leisure time and may also seek opportunities to engage with the broader community (15). This requirement is particularly pronounced among rural older adults (27).

We identified nine studies that address the cultural and recreational needs of older adults through digital technology applications.

Several studies have focused on utilizing digital technology to enhance the cultural and recreational experiences of rural older adults through community-based initiatives. Evidence suggests that older adults seek more recreational facilities (28). In response, Yujing Gu (19) proposed the deployment of sound equipment in community squares to support square dancing activities, integrating it with a smart short-video system to record and share moments of participation. This system allows older adults to view or disseminate videos on social media by scanning Quick Response (QR) codes (19). Similarly, Yangming Hu et al. (17) evaluated Lehoo Home, a comprehensive senior living center that employs an older adult profiling system to collect data and develop personalized leisure and care plans tailored to the specific needs of its residents.

Other studies have extended beyond community settings to explore the potential of digital platforms in providing a broader range of recreational opportunities. One example is the healthcare-integrated smart older adults care grassroots service system developed in Zizhou County (22). Additionally, Weiwei Wei and Rui Zhu (27, 33) have proposed the use of smart senior service platforms. Weiwei Wei’s platform integrates online and offline activities, offering services such as remote interactions, video communications, and chess games through transmission devices (27). In contrast, Rui Zhu’s platform creates interest groups and patient communities using user data, facilitating discussions on health conditions and recreational interactions, while also enabling offline social activities (33). Both studies underscore the significant role of digital technologies in promoting social engagement and improving the quality of life for older adults.

Beyond intelligent platforms, bespoke technologies present a novel approach to supporting the aging population. The Internet of Things (IoT) has enabled the development of sense-and-control convergence platforms as well as data-sharing and exchange systems to address the social needs of older adults (15). For instance, Lichun Yang developed entertainment robots that provide activities such as singing and dancing (20). These technologies complement platform-based solutions by addressing specific aspects of older adults’ well-being.

## 4 Discussion

To address the first two research questions, namely, the current application of digital technologies for aging to the rural areas of China and the needs of rural users for smart aging, the digital technologies elucidated from the systematic review were categorized according to four types of needs: (1) Addressing the daily care needs of older people; (2) Addressing the healthcare needs of older people; (3) Addressing the spiritual comfort needs of older people; and (4) Addressing the cultural and entertainment needs of older people.

### 4.1 Analysis of the frequency of use of digital technologies in rural areas

The data demonstrate that the frequency of utilization of digital technologies by rural older individuals exhibits considerable variability. Mobile devices and terminals are referenced in approximately 80% of the studies, followed by intelligent older adults care platforms/systems (73%), smart apps (33%), and a minor proportion of “virtual reality” or other unspecified technologies. This situation may be attributed to the accessibility of these technologies and the varying degrees of digital literacy exhibited by the rural population.

Considering the first research question, 33% of the studies in rural areas mentioned the use of smart apps for older adult care. These apps are typically connected to mobile devices or sensors to monitor body indicators and provide medical reports, medication reminders, and health education. Designing apps that are older adult-friendly and barrier-free is essential (37). However, owing to the low level of education among rural older adults, they may require assistance from guardians or family members to use these apps. Although not always necessary, assistance from guardians or family members can provide more comprehensive care and support (22). They typically have a better understanding of the situation’s needs and can provide prompt help. In cases where urban and rural zones are far apart, empty nesters living alone can use the app to remotely monitor the older adult’s lives. This enables timely interaction and care, fostering closer family ties between parents or guardians and their older loved ones.

Approximately 73% (73.02%) of the studies mentioned the provision of services to rural older adults through intelligent older adults care platforms/systems that make comprehensive use of sensor devices, GPS, IoT, cloud computing, mobile devices, and other technological means (39), and contain information management platforms, intelligent home systems, emergency call systems, remote care systems, back-office monitoring systems, and older care information dissemination systems (21). The construction of the platform involves the exchange of data between service demanders and providers and sharing them with providers, such as medical institutions, senior care service organizations, and rural commercial service facilities through smart devices and mobile terminals. Rural healthcare capacity is limited owing to outdated diagnostic equipment and a shortage of experienced healthcare providers and qualified doctors (34). Additionally, many rural areas are geographically isolated. The aforementioned platform systems can partially overcome time and space constraints by providing telemedicine and remote guidance services (40).

Approximately 80% of the studies were related to mobile devices and terminals designed for older people. Yuechi Lu proposed various smart devices to cater to different needs of older adults (35). These devices are typically linked to an application, platform, or system, and can exchange data and information to provide safety and medical care services for older adults (24). The device currently includes physiological detection and warning features, such as heart rate, sleep, blood pressure, body temperature, and other physical indicators. It is also capable of conducting emergency functions, including location positioning, emergency assistance, and fall prevention. Additionally, it has auxiliary functions, such as schedule reminders, medication reminders, and exercise and health reminders (26).

There are a number of “virtual reality” or other unspecified technologies, which are presented in Supplementary Table 1. Five studies on the future of smart aging present specific design schemes. These studies provide ideas for addressing the problem of rural smart aging. Although the results of these studies are not entirely innovative, the subsequent challenge is bringing these ideas to market (27).

### 4.2 Extent to which digital technologies address the different needs of older people

Considering the second research question, out of the 26 studies analyzed in this review, 65% of smart senior living applications were found to provide basic life care services for older adults, whereas 96% of them could provide essential medical care and safety services. However, only 35 and 42% of the applications could cater to the needs of older adults for leisure and recreation and spiritual comfort services, respectively. These results indicate that senior care services in rural areas are unbalanced, with a greater need for cultural, recreational, and spiritual comfort services in addition to medical and safety care services (40). The issue of older adults care in rural areas in China is more prominent than in towns. Currently, the older adults in rural China have limited access to services, wherein most only receive basic life and medical care. However, many of these older adults are widowed or have children who are not present owing to work or living situations, leaving them in need of emotional and psychological support. Unfortunately, most available services do not meet these needs (41).

There is a “silver digital divide” among rural older adults, which is an insurmountable digital cognitive barrier owing to a lack of understanding of information technology among older people. This is especially true for older people whose physical functions have deteriorated and whose ability to learn new things is limited. Smart devices have not been designed with the special needs of the rural older adult in mind, resulting in low usage rates among this demographic. This has become a significant constraint to the development of smart aging technologies in rural areas, particularly considering the relatively weak economic foundation of China’s rural areas. The field of smart aging technologies in rural areas is still in its infancy, wherein the academic exploration of the topic is less than 10 years old (42). There is a single type of smart home care service; however, a certain degree of inconsistency exists between the demand for older adults care services and the current supply. Thus, meeting the diverse needs of older adults in the rural community is challenging (25).

Notably, progress has been made in the application of smart aging technology in rural China. However, systematic analyses of smart aging are yet to be realized, and gaps still exist in development research, particularly in rural areas (27). In addition to the material needs of the rural older adults, their mental health and social interactions also require urgent attention. The future development of smart aging technologies in rural areas should focus on introducing more cultural, artistic, and recreational elements to provide a wider range of activities. Additionally, facilitating connections among older people through means, such as virtual communities and online social networking platforms, can be beneficial.

### 4.3 Future research trends in the use of digital technology for rural older adults

Considering the third research question pertaining to the future development of rural smart older adults platforms/systems, older adults residing in rural areas often have limited financial resources, a lower level of education, and weaker technological skills. They may also have limited experience using smart wearable devices, mobile terminals, and the internet, resulting in a digital divide. To some extent, the direct participation of older adults in smart services is hindered, and some can only indirectly participate through their guardians or others (22). Additionally, the characteristics of the older adult population necessitate the retention of traditional manual services rather than complete replacement by new technologies (24). The improvement of the aforementioned platforms requires participation from the government, market, and society (40).

As digital technology continues to evolve, it is crucial to adopt a human factors/ergonomic and human-centered approach in the development of technologies, particularly those intended for older adults. A human factors/ergonomic approach ensures that technology is designed to fit users’ physical and cognitive abilities, promoting ease of use, safety, and comfort (43). First, it ensures that the technology is accessible and usable by older adults, who may have varying levels of physical and cognitive abilities. Incorporating ergonomic design principles can reduce the risk of injury or strain, thereby enhancing the safety of the technology. Moreover, a human factors/ergonomic approach considers the mental models and cognitive load of older adults. Technologies should be intuitive and require minimal learning effort, ensuring that users can easily understand and interact with them. Feedback mechanisms are crucial, providing users with immediate responses to their actions, thus enhancing their confidence and satisfaction in using the technology (44).

In rural areas, hierarchical intelligent terminal equipment must be provided for older adults based on their self-care situations. Those with a higher degree of self-care can be issued a machine free of charge, whereas empty nesters, older adults living alone or under semi-self-care, and conscious older adults can be equipped with a key pager. For those with dementia or other poor mental states, smart bracelets should be issued (42). To improve conditions for rural older adults, high-end equipment terminals, such as smart doors, smart beds, and smart TVs can be installed (16). However, the economic situation of the family must be fully considered when introducing these smart facilities. Emergency alarm facilities and home security devices should be prioritized for rural families as they are necessary. Other types of intelligent devices should be chosen based on the family’s financial resources. Notably, older adults may have lower acceptance and learning ability when operating new devices. Therefore, devices with simple operation, easy interaction, and high recognition must be chosen for their benefit (35). The design of intelligent terminal devices should focus on light operation and intelligence (31).

As digital technology advances, it is crucial to consider the specific needs and limitations of older adult users, especially in rural areas. Future research and development should focus on adopting an ergonomic, human-centered approach to ensure that technological artifacts are accessible, usable, and beneficial for older adult individuals.

The current state of rural development is unique, and the smart aging field has several limitations. Implementing smart aging technologies in rural areas, multiple initiatives are necessary. The specific applications in this field can be classified into three principal branches: smart communities/rural, intelligent healthcare and smart home field. The concept of smart community is derived from smart city, which is a new concept and method of community management. Rural smart community integrates the use of big data, Internet of Things (IoT) and other modern technologies to create an intelligent field of social activities (45). In China, intelligent healthcare is defined as the application of a range of technologies, including smart healthcare products, information platforms, network technologies, intelligent control technologies, communication technologies, and cloud computing, to provide a variety of healthcare services. These include professional care, health testing and management, health risk assessment, reminders, and timely alerts (46). Smart homes equipped with integrated e-health and assisted living technologies represent a promising application of IoT in gerontechnology, potentially transforming healthcare systems for the older adults (47).

Analysis of the current application areas of smart aging in rural China reveals a need more research in the fields of smart healthcare and smart rural/community aging, whereas relatively few applications exist in the field of smart homes. This trend reflects the current development and limitations of smart aging technologies in rural China. In the field of smart healthcare, smart technologies have extensively been evaluated for medical monitoring, remote diagnosis and treatment, and health safety management. They can provide more convenient and timely healthcare services in rural areas to effectively address the shortage of healthcare resources and enhance the level of healthcare services and health management in these areas.

**Figure 3:**
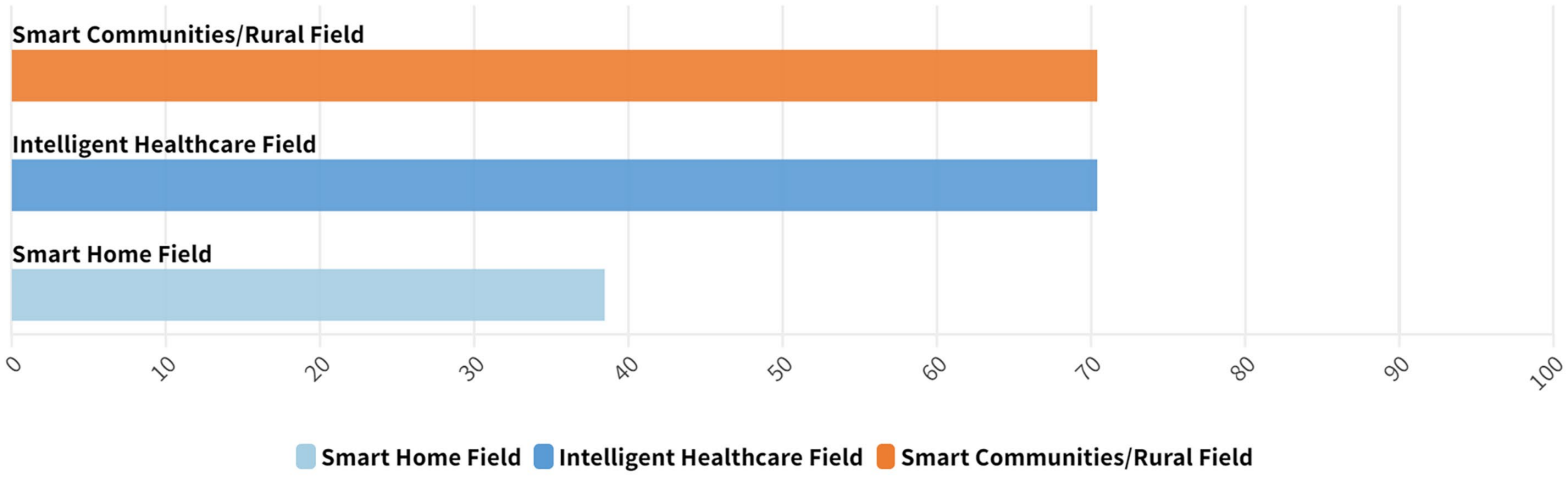
Distribution of literature across different domains of smart aging.

In smart rural aging/smart community aging, smart technology has improved the quality of life by providing community services, social interaction, and other support. This has compensated for the lack of social and living services through the construction of smart community platforms. However, the current application of smart home technology is relatively limited (Figure 3). This may be attributed to various factors, such as the need for greater promotion of technology, increased popularity, the unique situation of older adults in rural areas, and the acceptance of smart home devices in rural areas. In the future, the promotion of smart homes in rural areas will be facilitated by addressing the challenges of technology adoption and increasing awareness among the population. Despite significant progress in smart healthcare and smart aging in rural communities, smart homes still face challenges. Future research and practice should focus on innovating and promoting smart home technology to provide more comprehensive and diverse smart aging services that meet the multi-level requirements of rural areas.

### 4.4 Limitations

This study focuses on rural areas in China; however, geographical and cultural differences across regions may affect the applicability of the findings. For example, the acceptance of technology and the state of infrastructure can vary significantly between regions. Additionally, there is a tendency to emphasize the immediate benefits of smart aging technologies, such as improving healthcare and promoting independence, while often overlooking their long-term impacts and sustainability. Finally, rapid technological advancement means that findings can quickly become outdated. Continuous updates to the literature are necessary to remain aligned with new developments and emerging trends in smart aging technologies.

## 5 Conclusion

Rural China is experiencing a growing aging population, and smart aging technologies are seen as a solution to meet their increasing demands. As the population continues to age, the demand for smart aging technologies among rural older adults will also increase. Among the studies within the scope of this review, 74.07% assessed specific needs of rural older adults through questionnaires or theory. These needs include medical care and safety management, life care, spiritual comfort, and leisure and entertainment. The questionnaire survey revealed that besides the aforementioned needs, older adults also value their living environment and desire improved support facilities. Notably, China has made significant advancements in the application of smart older adults care in rural areas, particularly in terms of medical safety and basic life care. However, there is still significant room for improvement in current digital technology for smart aging to meet the leisure, entertainment, and spiritual comfort needs of older adults.

In cities, smart aging has shifted to specialized and high-tech solutions with economic growth, allowing older adults to live alone for longer periods of time (39). In Hong Kong, there is an initiative to create age-friendly living environments through the use of smart home technologies (SHTs) (48), which can provide interactive technology and unobtrusive support systems (49, 50). Shanghai’s smart aging initiatives rank among the most advanced in the nation, with technology as the forerunner and platform construction as the backbone, gradually building and promoting industrial demonstration projects (51). However, rural areas have lagged behind in terms of economic development, and some expensive technologies and services do not match the purchasing power of older adults, or perceived user-friendly features or operational difficulties lead to the refusal of technology by older adults (39). This is because the older population in rural or lower urban areas lacks the digital literacy to use complex and advanced technologies. Even with digital guidance from family members, the impact of digital literacy on the sense of access of the rural older adult population remains moderate. Older adults will be willing to use smart technologies if they are easy to use, reliable, and secure as they do not want to spend considerable time and effort in learning how to use the technologies (9).

Our findings indicate that rural older adults have specific needs that extend beyond medical and safety concerns, including desires for improved living environments and support facilities. Despite advances in smart aging technologies, current applications often fail to address these broader needs effectively, partly because of economic constraints and limited digital literacy among rural populations. The technological adoption between urban and rural areas underscores the need for policies that promote inclusivity and accessibility. To enhance the impact of smart technologies in rural areas, several policy measures should be considered: (1) Develop and fund training sessions to improve digital literacy among older adult rural residents, ensuring they can effectively use new technologies (52); (2) Support the creation and subsidization of cost-effective, user-friendly technologies tailored to the needs of rural seniors (53); (3) Invest in infrastructure improvements to support the use of smart technologies, such as enhancing transportation and accessibility (54); (4) Foster collaborations between government, technology providers, and local organizations to promote inclusive and practical technological solutions.

**Figure 4:**
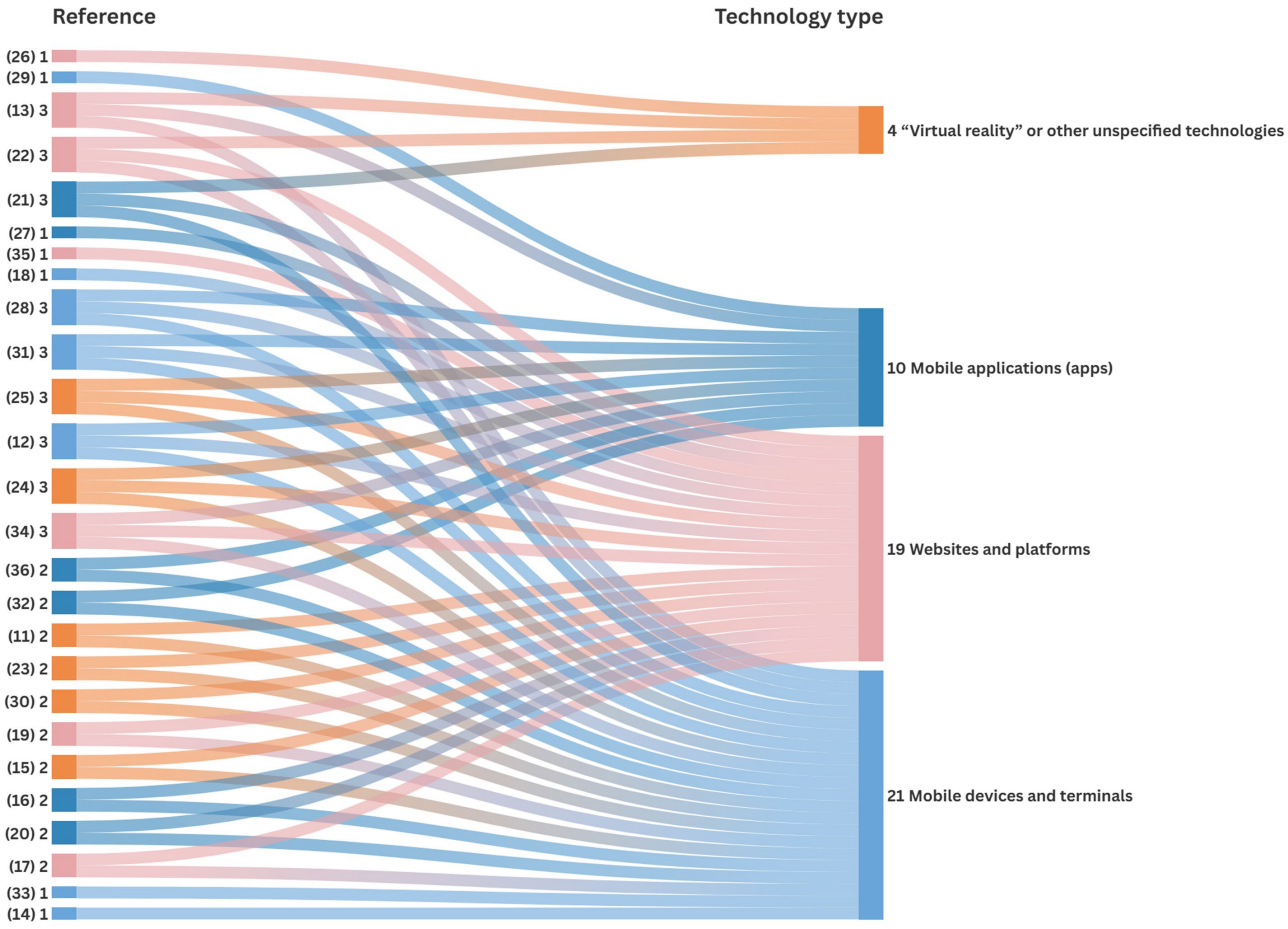
The number of digital technologies covered in the literature.

We present a systematic review of the main findings on digital technologies related to rural aging in China and explored the use of digital technologies to meet the diverse needs of older adults. These digital technologies were categorized into four groups (Figure 4): mobile applications (apps), websites and platforms, mobile devices and terminals, and VR technologies or other unspecified technologies (55, 56). Mobile applications (apps) are software solutions developed for a specific purpose (e.g., health monitoring or social interaction) and designed to run on smartphones, tablets and other portable devices. In contrast, mobile devices and terminals refer to the hardware, such as smartphones, tablets or wearable devices, that are used to access and interact with these applications. Our review indicates that while significant advancements have been made in medical safety and basic life care through smart technologies, notable gaps remain in addressing the full range of needs of rural older adults, including leisure, entertainment, and spiritual comfort. Digital technologies can provide rural older adults with convenient and timely medical monitoring, telemedicine, and life care services. Although some studies have explored recreational and spiritual needs, these aspects are often not addressed comprehensively. For example, while certain technologies cater to basic needs, there is a lack of advanced or tailored solutions for enhancing the quality of life through leisure and entertainment. This gap suggests that future research should not only continue exploring these dimensions but also focus on developing and evaluating interventions that specifically address these needs in more detail.

Rural areas often have inconvenient transportation services, lack age-friendly infrastructure, and have limited social and recreational resources compared to urban areas, potentially leading to prolonged periods of isolation at home. We found that digital technologies currently used in rural areas have limited applications in the realm of smart homes. Future research should investigate smart home older adults care services, which can aid in overcoming cognitive barriers to digital technology and enhance the understanding and acceptance of digital technologies.

Overall, the prospects for developing smart aging in rural China are promising. A thorough discussion of smart aging in rural China is expected to promote the comprehensive development of rural aging services, improving the quality of life of older people and addressing the challenges of an aging society. Thus, the entire society, including the government, social organizations, enterprises, and other parties, must collaborate in providing comprehensive and personalized older adults care services for rural villages. This will improve their quality of life and promote the innovation and application of digital technology in rural smart aging.

## Data Availability

The original contributions presented in the study are included in the article/Supplementary material, further inquiries can be directed to the corresponding author.
