# Supplementary material for: Digital technologies as solutions to China’s aging population: a systematic review of their opportunities and challenges in rural development
Source: Front Public Health. 2025 Jan 15;12:1416968. doi: 10.3389/fpubh.2024.1416968 (PMC11776298; doi:10.3389/fpubh.2024.1416968)
Supplement: Supplementary file 1 [file Table_1.pdf]

Supplementary Table 1. Digital technologies used by older people.

Reference (n=26)	Title	Technology	Technology type
(26)	Algorithm Model Design of the Aging Transformation Scheme of Computer Intelligent Aided Technology under the Background of Rural Revitalization	computer intelligent auxiliary technology	"virtual reality" or other unspecified technologies
(29)	Application of family-involved smart medication management system in rural-dwelling middle-aged and older adult participants with chronic diseases: Management of chronic diseases in rural areas	Smart Medication Management App	Apps
(11)	Construction of Smart Pension System for Urban and Rural Residents Based on Big Data Mining Algorithm	Intelligent Elderly(ec) Systems	Websites and platforms
		Wearable Devices	Mobile devices and terminals
(27)	Development of Elderly Care Industry in Urban-rural Areas in the Perspective of "Internet Plus"	Intelligent Health Detecting System	Websites and platforms
		Intelligent environmental control	
		Intelligent aided design	
(35)	Internet Hospital: Challenges and Opportunities in China	Internet Hospital Platform	Websites and platforms
(18)	New Rural Intelligent Pension Model Based on Big Data Technology	Pension Information Platform	Websites and platforms

(23)	Rural Smart Elderly Care Model: China’s Development and Challenges	Smart home systems and remote care systems	Websites and platforms
		one-touch connectivity to landlines and mobile devices, wearables and telemedicine devices	Mobile devices and terminals
(36)	The Comparative Study of Emotional Interaction Design of Empty Nesters in Urban and Rural Areas in China	Mobile phone App	Apps
		Intelligent robot, Smart bracelet	Mobile devices and terminals
(13)	Research on the Design of Future Retirement Communities in Villages From the Persperctive of Urban-Rural Integration	App	Apps
		NFC Smart Bracelet	Mobile devices and terminals
		AI Intelligent Display, AI Intelligent Sensing System, Intelligent Monitoring System	“virtual reality” or other unspecified technologies
(28)	A Case Study on the Governance of the Rural Concentrated Settlement Area of Dongyue Huayuan in Dayi County	"Yuju" APP	Apps
		Intelligent Recreation Application System	Websites and platforms
		Wearable Devices	Mobile devices and terminals
(30)	Research on Community Home Care Services for the Rural Areas in Zhoushan from the Perspective of Pluralistic Co-governance	Zhoushan Islands New Area Wisdom Senior Citizen Service Center's physical platform	Websites and platforms
		"Senior Citizen One-Click" cell phone, intelligent facilities, mobile terminal	Mobile devices and terminals
(32)	Smart Medical APP Design for Rural Empty	Smart Medical App	Apps

	Nest Elderly by General Practitioners	Smart Bracelet	Mobile devices and terminals
(19)	Research on the Improvement of Home-based Smart Elderly Care Services in Fuping County	Home Smart Aging Service System	Websites and platforms
		Smart Watch	Mobile devices and terminals
(31)	Research on Indoor Environment Construction Methods in Cold Areas Based on the Health Needs of the Elderly	APP	Apps
		Smart Elderly Service Integration Platform	Websites and platforms
		Smart Bracelet	Mobile devices and terminals
(15)	Study on Rural Ageing Construction under the Background of Seasonal Migration and Pension——Take Wuzhishan, Hainan Province as an example	Intelligent Elderly Platform, Intelligent Elderly Grassroots Service System	Websites and platforms
		Wired Emergency Caller, Automatic Sensor, GPS Locator	Mobile devices and terminals
(16)	Research on Zizhou County's Medical and Nutritional Integration Intelligent Elderly Care Model in the Context of Healthy China	Medical and Nutritional Integration Intelligent Elderly Grassroots Service System	Websites and platforms
		Smart Wearable Devices, Video Devices, Mobile Terminals	Mobile devices and terminals
(22)	Research on the construction of high-quality service system for rural intelligent elderly care in S County, Jiangsu Province	Open integrated business platform for smart aging, high-quality integrated supervision platform for smart aging	Websites and platforms
		Intelligent mobile devices or sensorless protection devices	Mobile devices and terminals

		VR technology, three-dimensional technology and integrated display technology, wireless positioning technology and short video technology	“virtual reality” or other unspecified technologies
(33)	Research on the Suitable Ageing Reform of Xiangxi Rural Residence under the Model of Home-based Care for the Aged	Intrusion sensing devices, detection devices, sensing devices, emergency one-button alarm devices, cell phones or other smart devices	Mobile devices and terminals
(25)	Research on the problems and Countermeasures of "hollow" village endowment insurance——Take Zhoushan as an example	App	Apps
		Zhoushan City "Smart Elderly" Service Platform	Websites and platforms
		Wearable Devices, Elderly Machines "One Click"	Mobile devices and terminals
(20)	Research on the difficulties and Countermeasures of carrying out intelligent home-based elderly care service in rural urbanization community ——Take Zhangjiakou E rural community as an example	Wuzhen Intelligent Aging-in-Place Service Center	Websites and platforms
		Bracelets, wristwatches, blood pressure monitors, monitoring equipment, smartphones	Mobile devices and terminals
(17)	An Initial Exploration of Smart Elderly Care Models in Rural Areas	Elderly information platform, service operation platform and management platform	Websites and platforms
		One-touch pager, smart bracelet, senior phone	Mobile devices and terminals
(12)		App	Apps
		Smart Home Elderly Service Platform	Websites and platforms

	Research on Rural Intelligent Home Nursing Service Model-Taking Qingshui County in Gansu Province as an Example	Smart Bracelet, Smart Watch, Smart Door, Smart Bed, Smart TV	Mobile devices and terminals
(24)	Research on the Service Model of Smart Elderly Care in Rural Areas——Based on the Investigation of Two Villages in South Shanxi	App	Apps
		Rural Smart Elderly Service Platform	Websites and platforms
		Bluetooth bracelets, ISO devices, specialized handheld devices, gravity sensors, smart pagers	Mobile devices and terminals
(21)	Feasibility study on the development of rural Shared pension mode under the situation of "separation of three rights"	Shared Aging Big Data Information Platform	Websites and platforms
		Smart Mobile Devices	Mobile devices and terminals
		Internet Information Technology and Sensors	“virtual reality” or other unspecified technologies
(34)	Here's how the picture of the digital countryside unfolds	APP	Apps
		Digital Countryside Cloud Platform	Websites and platforms
		Smart Watch, Arrangement of Smoke Sensors, Door Magnetic Alarms	Mobile devices and terminals
(14)	Research on the Application of Smart Elderly Products in China's Rural Home Care	Smartwatch, Bracelet, 360 Smart Camera, Portable ECG, Wireless Sensor	Mobile devices and terminals
